# Bibliometrics Analysis of Diagnostic Test Accuracy Studies of Bladder Cancer

**DOI:** 10.1155/2021/8870353

**Published:** 2021-08-13

**Authors:** Yuan Zhang, Yao Xu, Shuo Li

**Affiliations:** ^1^School of Foreign Languages, Wuhan College of Arts & Sciences, No. 1 Shenghai Boulevard, Huangpi District, Wuhan, China; ^2^Department of Laboratory Medicine, Zhongnan Hospital of Wuhan University, 169 Donghu Road, Wuhan 430071, China

## Abstract

**Background:**

Bladder cancer is amongst the top 10 most common neoplasms worldwide. It is responsible for over 150,000 deaths per annum. It is of great importance to study its clinical diagnosis and management. As a literature synthesis technique, bibliometrics analysis helps us to take our knowledge of bladder cancer diagnosis research, topics, and trends a step further. It is critical to elucidate the literature landscape and its pertinent impact on this field, in order to have a better understanding and improved management of bladder cancer.

**Materials and Methods:**

Search terms related to bladder cancer diagnosis were used to retrieval publications which met the predefined criteria in the Scopus database developed by Elsevier. Included articles were further evaluated by year of publication, country, language, institution, article type, source journal, coauthorship networks, and text mining of titles. The R software, as well as the *tm*, *ggplot2* packages, and the VOSviewer (version 1.6.15) were used as software tools.

**Results:**

A comprehensive literature search in the Scopus database returned a total of 824 publication items. Among them, 601 (72.94%) were published as journal articles, and 117 (14.20%) were published as reviews. The number of annual publications in this field has been increasing in recent years. English represents the language used in the majority of the articles (634, 77.13%). The highest number of citations received for a single article is as high as 628 times. We also clustered and visualized a network of 701 authors with no less than 3 publications. The term cloud and hierarchical clustering dendrogram which revealed with text mining were used to discover the hot research topics in this field. We also statistically analyzed evolution of the major journal impact indices, including Impact Factor, EigenFactor Score, and CiteScore of 11 journals with a period of 9 years.

**Conclusion:**

Through bibliometrics analysis, we found hot topics and related research focuses and trends in bladder cancer diagnosis. This bibliometrics analysis has identified influential articles in the field and provides a useful guide to researchers as to what type of article constitutes a highly citable publication in this subject. In addition, a coauthorship network helps researchers find out which team may be a potential partner and where their studies focus on.

## 1. Introduction

Bladder cancer (BLCA) accounts for 212,536 deaths and 573,278 new cancer cases worldwide in the year of 2020, according to the latest report of global cancer statistics [1]. BLCA is the sixth most common cancer and the ninth cancer for mortality in males worldwide, and it is not among the top ten cancers for incidence and mortality in females [1]. Established risk factors for BLCA include tobacco smoking, professional hazards exposure, bladder stone, bladder infectious diseases, and chronic cystitis; however, many patients were absent for these risk factors [2]. When it comes to the diagnostic methods currently in practice for bladder cancer, cystoscopy and urine cytology are two important procedures. Cystoscopy is an invasive tool and has low sensitivity for carcinoma in situ, while urine cytology is a noninvasive and low-cost method which however has a high specificity but low sensitivity for low-grade urothelial tumors [3]. Despite continuous efforts in the search for liquid biopsy biomarkers, only a few biomarkers have been applied at present in daily clinical practice for the early and noninvasive detection of bladder cancer [3]. In-depth and comprehensive understanding of bladder cancer diagnosis is an important topic for researchers. As a literature synthesis technique, bibliometrics analysis helps us to take our knowledge of bladder cancer diagnosis research, topics, and trends a step further. Therefore, it is necessary to carry out bibliometrics to sort and analyze the published literature to find which hot topic is the key research point.

Bibliometrics takes the global literature landscape and document characteristics as the research object and uses methods such as mathematics and statistics to study the distribution pattern, quantitative relationship, and changing laws of document information and then to explore the structure, characteristics, and laws of science and technology [4]. Through bibliometrics research, analysis of literature-type distribution, source journals, citations, coauthorship network analysis, and text mining could help researchers to further understand the relevant fields [5]. A small number of bibliometrics analyses have been published in the field of urology, including andrology and urological emergencies. To the best our knowledge, none have specifically focused on bladder cancer diagnosis. Accordingly, in order to fill this gap, we carried out, in this study, a systematic bibliometrics analysis of the literature from the perspective of diagnosis focusing on bladder cancer.

## 2. Review on Previous Literature

According to our systematic review in the Clarivate Analytics Web of Science (WOS) database, there was no bibliometrics analysis regarding bladder cancer diagnosis (latest search update: June 10^th^, 2021). Mainwaring and his colleagues analyzed the top 100 most cited articles in a pool of 47,381 bladder cancer-related publications [5]. According to their results, the median number of citations among these top 100 was 515. The *Journal of Urology* published the greatest number of articles (*n* = 15) among these top 100. The most prevalent theme was the pathobiology of bladder cancer (*n* = 37), followed by oncological treatment (*n* = 17) [5]. Another previous study conducted a comprehensive bibliometrics summarization of highly cited articles that published spanning half a century on urological malignancies [6]. The authors conducted a PubMed search for articles published in the 13 most cited urological journals between 1955 and 2009. Of 97,554 articles published during this time, 1,239 articles were cited more than 100 times [6]. BLCA-related documents ranked second in terms of number of citations. In addition to the above studies, we also found some interesting studies of bibliometrics nature, within the scope of urological oncology but not precisely BLCA. For example, Kunath et al. discussed the role of gender effects in general oncological and uro-oncological research and represented the first bibliometrics analysis of its kind [7]. Despite the overall role of gender effect seemed small in general oncological research, it was increasing steadily. In uro-oncological research, such trend was also visible in bladder but not in renal cell cancer [7]. Although bladder cancer was also involved in these mentioned studies, none of them took bladder cancer diagnosis as the topic of interest. Consequently, a bibliometrics analysis of diagnostic test accuracy studies of bladder cancer is yet to perform in a focused and precise manner.

## 3. Materials and Methods

### 3.1. Overview of Study

In our bibliometrics analysis of bladder cancer diagnosis, we statistically analyzed the type of the documents and identified journals that published the largest number of manuscripts and/or the most cited manuscript. To find the collaboration partnership among authors and teams, we drew the coauthorship network. In order to discover the hot topics in the field of bladder cancer diagnosis, we generated the term cloud that included 196 most frequent terms. We also analyzed other essential bibliometrics parameters such as institutions, journals, and years of publication.

The aim of this bibliometrics analysis was to provide a useful guide to researchers in the field of bladder cancer diagnosis, to demonstrate what type of article constitutes a highly citable item in this subject, and to identify hot topics and research themes together with the most eminent and influential articles that have helped to further extend our knowledge and understanding of bladder cancer diagnosis. To analyze the dynamics of the related journal impacts, we also statistically analyzed evolution of the major journal impact indices, including Impact Factor, EigenFactor Score, and CiteScore of 11 journals for a period of 9 years.

### 3.2. Literature Search and Data Acquisition

**Figure 1:**
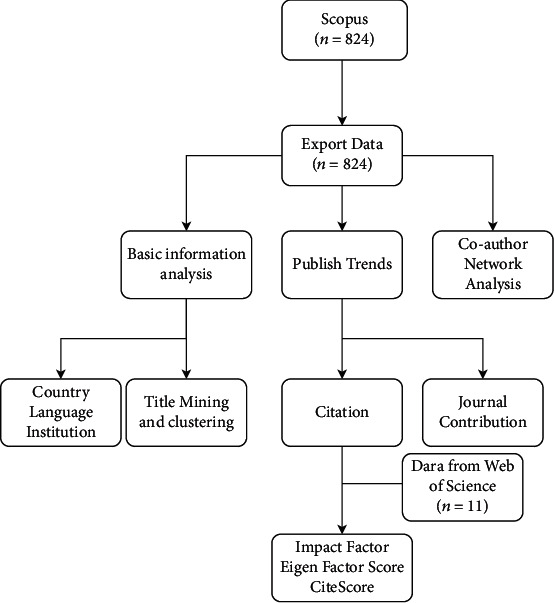
Workflow of the bibliometrics analysis.

In this study, the Scopus database under Elsevier was selected as the source of the literature information. Scopus holds more than 19,000 source journals from 4,000 publishers around the world and is the world's largest abstract and citation database, providing researchers with a one-stop platform for obtaining scientific and technological literature [4]. Scopus also provides a variety of convenient retrieval methods and download paths. All publications no later than 31 Jan 2021 were considered. In order to obtain literature related to the diagnosis and testing of bladder cancer, in the search strategy, the search range was set to “title,” and the keywords were limited to the combination of “bladder” and “cancer” and “diagnosis.” The search formula used is as follows: (TITLE (bladder) AND TITLE (cancer) AND TITLE (diagnosis)). The search results were exported in CSV and RIS file formats, which included information on authors, year of publication, title, and source journal among 25 items in total. In addition, we extracted the journals' Impact Factor and EigenFactor Score data of several journals from the WOS database. All data in this research could be found in the Scopus and WOS database. Workflow is shown in Figure 1.

### 3.3. Statistical Analysis and Visualization

We took bibliometrics as the primary study approach to analyze the relevant literature on the diagnosis of bladder cancer to form an overview and summarization of the related literature landscape. We drew a time distribution map of the literature published over the past about 70 years and analyzed the distribution of countries, institutions, and journals. In addition, we also looked at where those documents came from and which journal paid more attention on bladder cancer diagnosis. In order to show part of journal analysis and institutions analysis, we carried out the statistical analysis of the top ten journals and institutions in terms of the number of publications. The statistical analysis of citations could help us find the subset of documents making greater impact and contributions to bladder cancer diagnosis research. Then, we calculated the number of citations per article and used it as an index to evaluate the influence of the literature. Besides, we created a table showing the top ten cited publications. We created and visually displayed a network of coauthors composed of authors who have published no less than 3 articles, where the coauthoring relationship between the authors of a certain document was considered. In order to find more precise research hotspots of the bladder cancer diagnosis, we performed text mining of all articles' titles. We also created hierarchical clusters based on the relevance of the words in the titles. The more times the word appears, likely the more important the word is in bladder cancer diagnosis research. We selected 196 keywords with the highest frequency to construct the term cloud to show the hotspots of bladder cancer diagnosis research, which could help the community identify where the cutting-edge topics locate.

In addition to the bibliometrics analysis above, we also carried out the analysis of Impact Factor, CiteScore, and EigenFactor Score [4]. These statistical indicators are considered reliable bibliometrics measurements for journal impact, particularly in terms of the Impact Factor which has received a lot attention from researchers, editors, and librarians and is widely used for the assessment of research and journal significance [4]. But there are also a lot of people who think that we must move away from an over-reliance on journal IF to seek new ways of assessing research output [4, 8]. CiteScore is a new standard that provides a more comprehensive, transparent, and current view of a journal's impact, and it is also popular in databases such as Scopus. EigenFactor Score uses a complex algorithm, similar to Google's Page Rank, which takes into account not only the number of citations but also their “quality” by assigning specific weights to the source of the citations [4].

### 3.4. Software and Version

We use R software 3.6.3 to carry out the text mining work, cluster the title words, and draw the cloud word. Briefly, we use the “tm” package to carry out the program of mining text of the titles and the “ggplot2” package to visualize the result of clustering and cloud word. The VOSviewer 1.6.16 software was used to visualize collaboration between authors in the form of coauthorship network [9]. In addition, several tables in this document were generated in the Microsoft Excel program.

## 4. Result

### 4.1. A General Overview of the Literature

Accessibility to Scopus, the largest abstract and citation database in the world, was obtained through Wuhan University library. And the search time range was set to before February 2021. The search term was limited to include “bladder,” “cancer,” and “diagnosis,” and the search range was set to “title” only. All search results were exported as tables in the CSV format. And the RIS document style was exported for the coauthor network analysis using VOSviewer software.

We retrieved a total of 824 documents related to bladder cancer diagnosis. Among them, 601 (72.94%) papers were published as articles, 117 (14.20%) were published as reviews, and the rest of papers were published in other types. The documents involved 53 journals and 701 researchers from more than 160 affiliations in 57 countries/regions.

### 4.2. Time Distribution of Publications

**Figure 2:**
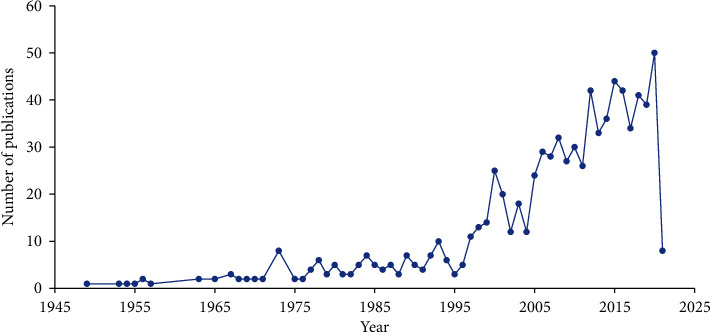
Trends of number of publications.

The literature on the diagnosis and testing of bladder cancer has developed over time, as shown in Figure 2; it can be seen that the number of literary publications has shown an upward trend over the past few decades. Although the growth rate fluctuates, the overall growth trend is obvious. It achieved the first rapid increase in the number of publications around 1973, and then the number grows rapidly and steadily after 1995. Up to the time of retrieval of those documents, 824 articles have been collected. We statistically calculated that the compound growth rate from 1988 to 2019 was as high as 7.55% (Figure 2).

### 4.3. Country, Language, and Institution

Five countries have posted no less than 50 articles: United States (*n* = 186), China (*n* = 95), Germany (*n* = 92), United Kingdom (*n* = 70), and Japan (*n* = 50). There are 29 countries that have posted no less than 5 articles in this field. The United States has the greatest research achievements in this field, which is in line with the reality of the development of bladder cancer diagnosis.

English is the most used language for all documents published for the first time, with 634 (77.13%) articles. Next is Russian, with 46 (5.60%) articles. There are six languages which were used as the primary publishing language in more than 10 papers, English (*n* = 634), German (*n* = 32), Japanese (*n* = 30), Russian (*n* = 46), Chinese (*n* = 28), and Spanish (*n* = 18). In total, more than 20 languages appeared in the documents that we retrieved in Scopus.

The top ten institutions in terms of the number of publications are shown in Table 1. Most of these institutions are world-renowned research institutes, with prominent positions in the history of cancer research and development [5]. Focusing on the leading institutions around the world allows us to understand better about the progress of bladder cancer diagnosis.

### 4.4. Journal Distribution

The retrieved documents involved a total of 153 journals or academic conference proceedings, with an average of 5.4 articles per journal. In order to show the approximate range of the number of published documents, the journals which published no less than 9 documents are shown in Table 2. These journals published a total of 195 papers related to the diagnosis and testing of bladder cancer, accounting for 23.66% of the total literature pool. The journal with the largest number of publications is the *Journal of Urology* (42 documents, 5.10%), which is much higher than the second-ranked *European Urology* (29 documents, 3.52%) journal. Among these journals, 8 out of the top 12 journals are of urology professional, and the current highest Impact Factor (2019/2020) is with *European Urology* (IF = 17.95). In the rest of the journals, most (*n* = 113, 73.86%) published less than 3 articles.

### 4.5. Citation

570 of 824 retrieved documents have been cited for at least one time, accounting for 69.17% of the total documents, and the total number of citation times was 14,200. Among them, nearly half of the documents (*n* = 276, 48.42%) were cited for less than 10 times, and 971 citation events came from them. The average number of citations of the cited documents is 24.91. The basic information of the top 10 most cited documents is shown in Table 3. The highest number of citations for a single article is as high as 628, and there are two documents with more than 300 citations. It is interesting that the top ten most cited articles were not published in the journals with the largest number of publications (Table 2).

**Figure 3:**
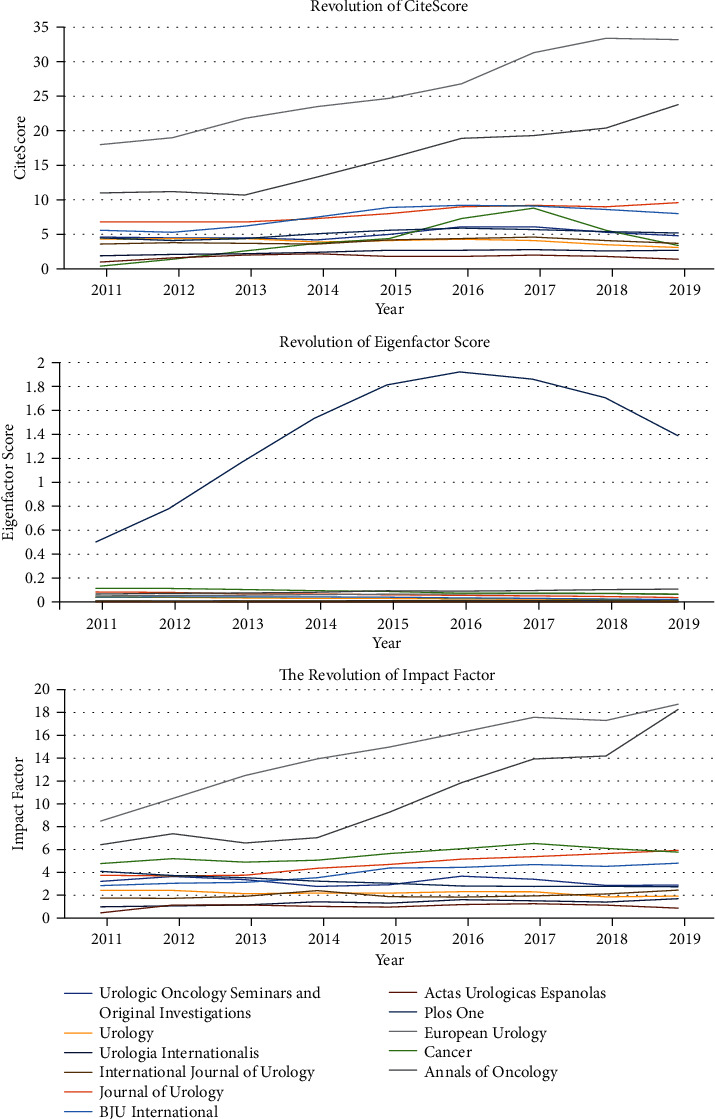
The evolution of IF, ES, and CS of 11 journals which published ≥9 papers related to bladder cancer diagnosis.

As to the evolution of the Impact Factor, EigenFactor Score, and CiteScore, we drew a combined line graph (Figure 3) demonstrating the indices' historical evolution across a 9-year period for 11 journals which published over 9 papers in the past 70 years. We noted that the evolution trajectory was not dramatic for most of the journals for these measurements, with a few exceptions that two journals, *European Urology* and *Annals of Oncology*, showed a predominant increase in both Impact Factor and CiteScore compared to other journals, and one journal Plos One showed apparently different performance in terms of EigenFactor Score.

### 4.6. Researcher and Coauthor Network Analysis

**Figure 4:**
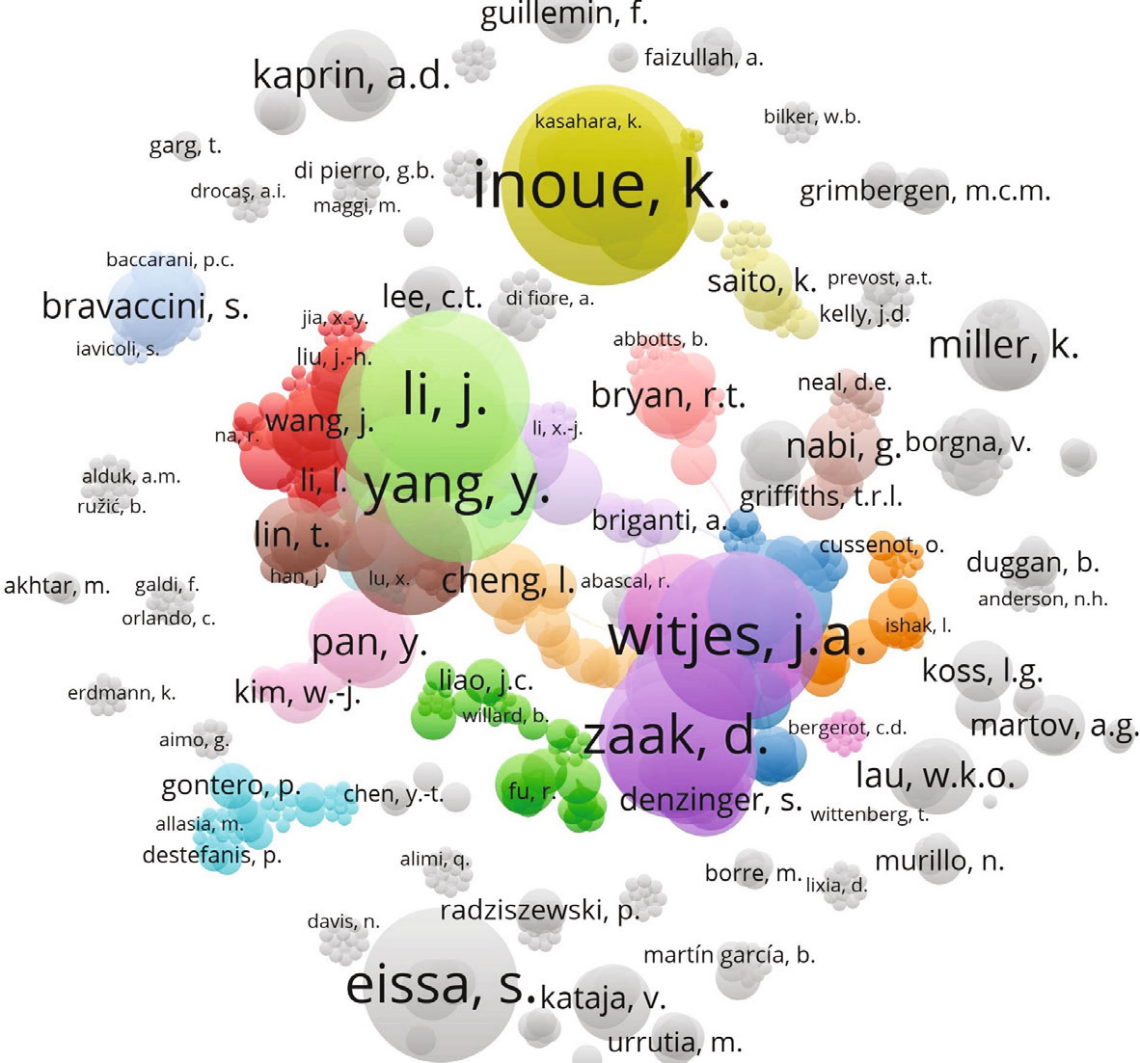
The clustering visualization for 701 authors' coauthorship network.

According to statistics of the documents, a total of 701 authors, signed 4,405 times, have participated in the research of all 824 articles, with an average of 5.3 researchers participated in each article. Among all researchers, Doctor Keiji Inoue from the Department of Urology of Kochi Medical School published the largest number of documents, up to 12 papers. We clustered and visualized a network (Figure 4) of 701 authors according to that if they have participated in cooperation in one document. Each node in Figure 4 represents a researcher, and the connection between two nodes represents the coauthoring relationship between two researchers. The size of the node is proportional to the number of articles published by the corresponding researcher. Based on the coauthoring relationships among 701 authors, clustering analysis separated them into 74 subgroups, and the largest cluster involved as many as 73 researchers. Each subgroup corresponds to a closely cooperating research team, which was marked in different colors (Figure 4). It can be clearly found that the cooperation in each researcher team is very close, and the cooperation between different teams within a given network cluster is also frequent but much less between clusters. It seems that the cooperation between various teams around the world in the diagnosis and testing of bladder cancer is not so close as we thought.

### 4.7. Title Information and Text Mining

**Figure 5:**
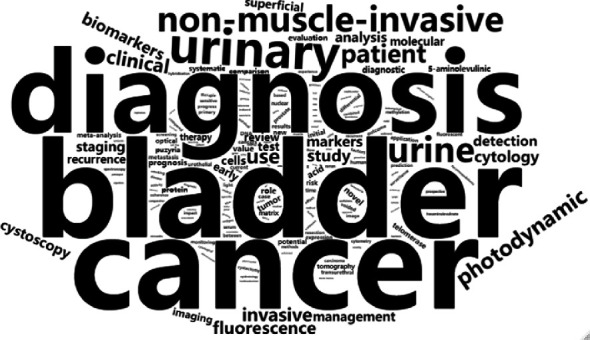
The term cloud of the top 196 most frequently occurred terms.

Text mining was conducted on the titles of 824 documents related to the diagnosis and testing of bladder cancer. Based on the preprocessed text corpus, there are 2,500 entries, 100% sparsity, and entry-text matrix with a maximum entry length of 60 bytes. The top five frequent terms are “bladder” (855), “cancer” (853), “diagnosis” (804) “urinary” (228), and “non-muscle-invasive” (94). A total of 2,300 entries had no more than 10 occurrences, accounting for 92.00% of all entries. We generated word cloud for words that occurred in no less than 5 titles, totaling 196 terms, suggesting a long-tailed distribution of term frequency. In the term cloud (Figure 5), the font size of an entry is directly proportional to the frequency of the entry. We can clearly find the high-frequency entries. This visualization could help us find which topics in this field has been studied many times.

**Figure 6:**
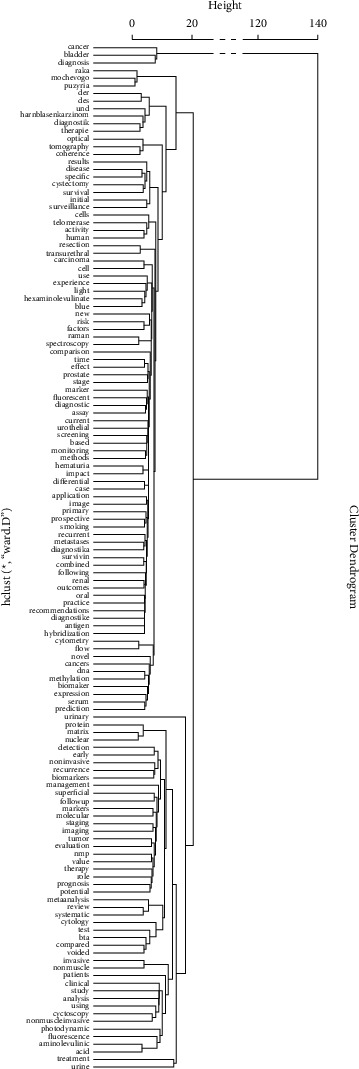
Hierarchical clustering dendrogram of the top 124 entries with correlation.

After association analysis, it is determined that the term with the highest degree of correlation with “bladder” is “cancer” (*r* = 0.36, correlation coefficient); then, the entries with the highest correlation degree are “mucosa” (*r* = 0.27), “cytology” (*r* = 0.26), “antigen” (*r* = 0.26), “lavage” (*r* = 0.19), “resection” (*r* = 0.14), and “biopsies” (*r* = 0.13), among others. We performed hierarchical clustering analysis on the top 123 entries with high correlation and drew a dendrogram (Figure 6). Roughly clustered into five categories based on term frequency and relevance. Combining the contents of Figures 5 and 6, readers could have a quick and overview understanding of the distribution of hot research topics in this field.

## 5. Discussion

Bladder cancer is known for its high rate of recurrence and cost to treatment. Therefore, bladder cancer diagnosis is of great significance in medical research and clinical practice, particularly for urologists and bladder cancer patients [10, 11]. Diagnosis is related to the therapeutic timing and management strategy, so effective diagnostic methods have always been the primary subject in the field of bladder cancer clinical management. A lot of research reports and data summary analysis on bladder cancer diagnosis have helped researchers to form high-quality evidences, and systematic bibliometrics analysis could be used to guide not only cancer research design but also clinical practice [4, 12]. For the first time, we conducted bibliometrics analysis on the topic of research progress and literature publication in the diagnosis and detection of bladder cancer. In addition, to extract important information, we also carried out text mining and generated relevant visual display. Text mining could help find what are the important words in the studies and which topics are appearing frequently about the advancement in this area [13]. According to the analysis results of this study, the number of publications related to bladder cancer diagnosis and testing has been increasing in the past 30 years, and its growth is basically consistent with the increase in the overall number of medical research publications [14, 15]. The volume of literature related to the diagnosis and testing of bladder cancer published by American scholars ranks first amongst various countries, accounting for nearly one-quarter. In terms of journal distribution, we can clearly see that the literature on bladder cancer diagnosis and testing is concentrated in a few specific journals. Among them, the *Journal of Urology* has published 42 articles, followed by *European Urology*, 29 documents, in the second place. In terms of the number of articles published by a single scholar, Doctor Keiji Inoue, a Japanese researcher has published the largest number of documents. The citation of a document is an important indicator directly reflecting its academic influence [16, 17]. Through comparisons, we can see that the most influential literature is basically review articles from internationally renowned medical institutions, representing the few essential classic literatures on bladder cancer diagnosis and testing [18, 19]. The coauthorship network involving 701 scholars with adequate publication contribution in the field suggests that there is a close cooperative relationship between different research teams, and most researchers will participate in the research work of multiple research teams. However, the collaboration seems to be confined within team clusters. It can be seen that collaborations between scholars confer a great advantage in collecting more information for researching design and performance [17]. We speculate that the reason for this phenomenon is that the diagnosis and detection of bladder cancer are the core clinical problem, and its research requires a lot of scientific research investment and empirical analysis, and cross-disciplinary collaboration is needed to better conduct the research. The results of title information text mining show that except for the top five terms appearing frequently other key terms such as “treatment” (*n* = 92), “clinical” (*n* = 52), “invasive” (*n* = 49), and “cytology” (*n* = 42) which also represent the important research aspects associated with bladder cancer diagnosis. Term cloud and hierarchical clustering are important ways to find hot topics of bladder cancer diagnosis. More than half of the top ten journals in Table 2 are urological oncology journals. It can be inferred that these professional journals of urology are more easily recognized by researchers [20, 21]. Seven of the top ten highly cited documents also came from the top ten journals in Table 2, which published the most articles of bladder cancer diagnosis.

Bibliometrics analysis will provide valuable references and suggestions, especially for cancer diagnosis research. And it could offer us a landscape of the literatures in researchers' areas of concern, such as bladder cancer diagnosis methods and biomarkers. The methodology of bibliometrics analysis is always on living development, and there are many scholars that have developed bibliometrics methods in a systematic and comprehensive manner [4, 8, 22]. As a follow-up of the present study, we will keep an eye on the hot topics and their evolution in the field of bladder cancer diagnosis, and an updated bibliometrics analysis will be planned where significant new data are available. In the future research, on the other hand, a potential direction of interest is to classify the bladder cancer diagnosis literatures first, by diagnostic procedures such as those based on liquid biopsy, imaging, and emerging new biotechnology, and then conduct more focused bibliometrics analysis in combination with other literature review techniques such as meta-analysis.

The present study also has some limitations just like other researches. The topic is focused to bladder cancer diagnosis; thus, only a limited number of studies are included. However, the study can be efficiently used as a reference for further bibliometrics analysis for other cancer types. The analysis of the evolution of bibliometrics measurements of the journals' impact is currently limited to a more descriptive scale. It could be further extended to a comprehensive modeling as a future subjective, like previously described [8, 23].

## 6. Conclusion

The bibliometrics analysis of bladder cancer diagnosis has a great guiding role in the research of diagnosis and treatment research of bladder cancer. We summarized the characteristics of literature on bladder cancer diagnosis. We drew a landscape of publication year, affiliation, citation, source journal, coauthorship, and title text, revealing the key research hotspots. We demonstrated that the most popular research direction in this field is the early detection for non-muscle-invasive disease, which confirms the important position of bibliometrics analysis of diagnostic testing in clinical oncology research.

## Figures and Tables

**Table 1:** Institution distribution of publications related to bladder cancer diagnosis.

Sort	Institution	No. of publications
1	Ludwig-Maximilians-Universität München	15
2	Radboud University Nijmegen Medical Centre	14
3	UT Southwestern Medical Center	11
4	Kochi Medical School	10
5	Ain Shams University, Faculty of Medicine	9
6	Stony Brook University	9
7	Universität Regensburg	9
8	University of Texas MD Anderson Cancer Center	8
9	Sun Yat-sen University	8
10	University of Miami Leonard M. Miller School of Medicine	8
10	Charité–Universitätsmedizin Berlin	8
10	Qilu Hospital of Shandong University	8
10	Renaissance School of Medicine at Stony Brook University	8

**Table 2:** Journals published no less than 9 items.

Journals	No. of documents	Impact factor^∗^
Journal of Urology	42	5.925
European Urology	29	17.947
Urology	20	1.924
BJU International	18	4.806
Cancer	18	5.742
Urologic Oncology Seminars And Original Investigations	11	2.882
Urologiia Moscow Russia 1999	11	NA
Urologia Internationalis	10	1.698
Actas Urologicas Espanolas	9	0.873
Annals of Oncology	9	18.274
International Journal Of Urology	9	2.445
Plos One	9	2.74

^∗^Journal impact factor based on Thomson Web of Knowledge Journal Citation Reports Ranking (2019).

**Table 3:** Basic information of the top ten documents in total citations.

First author	Title	Year	Journal	Cited times	DOI
Kirkali Z.	Bladder cancer: Epidemiology, Staging and Grading, and Diagnosis	2005	Urology	628	doi:10.1016/j.urology.2005.07.062
Chang S.S.	Diagnosis and Treatment of Non-Muscle Invasive Bladder Cancer: AUA/SUO Guideline	2016	The Journal of Urology	380	doi:10.1016/j.juro.2016.06.049
Smith S.D.	Urine Detection of Survivin and Diagnosis of Bladder Cancer	2001	Journal of the American Medical Association	251	doi:10.1001/jama.285.3.324
Mourant J.R.	Spectroscopic Diagnosis of Bladder Cancer with Elastic Light Scattering	1995	Lasers in Surgery and Medicine	248	doi:10.1002/lsm.1900170403
Burger M.	Photodynamic Diagnosis of Non-Muscle-Invasive Bladder Cancer with Hexaminolevulinate Cystoscopy: A Meta-Analysis of Detection and Recurrence Based on Raw Data	2013	European Urology	234	doi:10.1016/j.eururo.2013.03.059
Denzinger S.	Clinically Relevant Reduction in Risk of Recurrence of Superficial Bladder Cancer Using 5-Aminolevulinic Acid-Induced Fluorescence Diagnosis: 8-Year Results of Prospective Randomized Study	2007	Urology	225	doi:10.1016/j.urology.2006.12.023
Yoshida K.	Telomerase Activity in Bladder Carcinoma and Its Implication for Noninvasive Diagnosis by Detection of Exfoliated Cancer Cells in Urine	1997	Cancer	206	doi:10.1002/(SICI)1097-0142(19970115)79:2<362::AID-CNCR20>https://3.0.CO2-Y
Glas A.S.	Tumor Markers in the Diagnosis of Primary Bladder Cancer. A Systematic Review	2003	The Journal of Urology	198	doi:10.1097/01.ju.0000067461.30468.6d
Pashos C.L.	Bladder Cancer: Epidemiology, Diagnosis, and Management	2002	Cancer Practice	198	doi:10.1046/j.1523-5394.2002.106011.x
Bellmunt J.	Bladder Cancer: ESMO Practice Guidelines for Diagnosis, Treatment and Follow-Up	2014	Annals of Oncology	192	doi:10.1093/annonc/mdu223

## Data Availability

The data source of this work was publicly available literature. Other data associated with the findings were available from the corresponding authors upon reasonable request.
